# Characterization of Biofilms Formed by Foodborne Methicillin-Resistant *Staphylococcus aureus*

**DOI:** 10.3389/fmicb.2018.03004

**Published:** 2018-12-04

**Authors:** David Rodríguez-Lázaro, Carlos Alonso-Calleja, Elena Alexandra Oniciuc, Rosa Capita, David Gallego, Camino González-Machado, Martin Wagner, Vasilica Barbu, José María Eiros-Bouza, Anca I. Nicolau, Marta Hernández

**Affiliations:** ^1^Microbiology Division, Department of Food Science and Biotechnology, Faculty of Science, University of Burgos, Burgos, Spain; ^2^Department of Food Hygiene and Technology, Veterinary Faculty, University of León, León, Spain; ^3^Institute of Food Science and Technology, University of León, León, Spain; ^4^Faculty of Food Science and Engineering, Dunarea de Jos University of Galati, Galati, Romania; ^5^Dependencia de Sanidad de Vizcaya, Delegación del Gobierno en el País Vasco, Bilbao, Spain; ^6^Institute for Milk Hygiene, Milk Technology and Food Science, University of Veterinary Medicine Vienna, Vienna, Austria; ^7^Department of Clinical Microbiology, University Hospital Rio Hortega, Valladolid, Spain; ^8^Laboratory of Molecular Biology and Microbiology, Instituto Tecnológico Agrario de Castilla y León, Valladolid, Spain

**Keywords:** MRSA, food, biofilm, biomass, confocal laser scanning microscopy, matrix

## Abstract

The objective of this study was to evaluate the capacity of 49 methicillin resistant *Staphylococcus aureus* (MRSA) from foods of animal origin (42 from dairy products and 7 from meat and meat products) to form biofilms. Overall, a higher biofilm biomass was observed for those MRSA strains harboring SCC*mec* type IV, while 8 MRSA strains (5 from dairy products and 3 from meat and meat products) were classified as strong biofilm formers in standard Tryptic Soy Broth medium. When a prolonged incubation period (48 h) was applied for those 8 MRSA strains, an increased biofilm biomass accumulation was observed during the time course, whereas the number of viable cells within the biofilms decreased as the biomass increased. The capacity of biofilm production correlated pretty well between the experiments using polystyrene microtiter plates and stainless steel micro-well plates, and significant higher values were observed in stainless steel when glucose was added to TSB during the enrichment. Biofilms were further characterized by confocal laser scanning microscope (CLSM), confirming that proteins and α-polysaccharides were the predominant components inside the extracellular polymeric matrix of biofilms formed by MRSA strains. In conclusion, our results confirm that MRSA isolates from foods of animal origin have significant capacity for forming biofilms with a high protein content, which can play a key role for the successful dissemination of MRSA lineages *via* food. Knowledge of the capacity of MRSA strains to produce biofilms, as well as characterization of the main MRSA biofilms matrix components, can help both to counteract the mechanisms involved in biofilm formation and resistance and to define more rational control strategies by using tailor-made cleaning agents.

## Introduction

Methicillin-resistant *Staphylococcus aureus* (MRSA) has emerged since antimicrobial therapy was introduced in hospitals, having nowadays the ability to resist several classes of antibiotics (Lowy, 2003). MRSA strains emerged in hospitals (Diekema et al., 2001; Enright et al., 2002), communities (O’Brien et al., 2004) and recently in foods and associated foodstuffs (Rodríguez-Lázaro et al., 2015). MRSA has recently attracted lot of attention in the area of food safety, as such strains may have a great impact on public health (Doulgeraki et al., 2017; Rodríguez-Lázaro et al., 2017). MRSA strains can form biofilms by adhering to different types of surfaces (e.g., indwelling medical devices) (Sadekuzzaman et al., 2015; Buzón-Durán et al., 2017). Recent studies showed that biofilms formed by *S. aureus* and MRSA strains may represent a hidden pathway for contamination of food and human handlers in food processing plants, by colonizing equipment and materials (Rode et al., 2007; Gutiérrez et al., 2012; Doulgeraki et al., 2017; Vergara et al., 2017).

Developing resistance to antimicrobial agents is similar to adopting survival strategies, which MRSA biofilms can benefit from Uršič et al. (2008). Gene expression is altered when MRSA adopts the biofilm mode of growth (Archer et al., 2011; Foulston et al., 2014) in response to different environmental changes such as temperature, osmolarity, pH, oxygen supply, source of nutrients and other factors that might appear (Costa et al., 2013; Srey et al., 2013). The increased metabolic rates may explain the capability of biofilms to act as diffusion barriers to slow down the infiltration of some antimicrobial agents (Archer et al., 2011). Singh et al. (2010) showed diminished penetration of antibiotics such as oxacillin, cefotaxime or vancomycin into biofilms formed by *S. aureus* and *Staphylococcus epidermidis*. However, those biofilms remained unaffected when amikacin and ciprofloxacin were introduced (Singh et al., 2010). Due to these reasons, the potential to cause infections in people with indwelling medical devices is highly increasing and as result, many studies have addressed this topic. However, the food sector remains an important niche that requires attention. From a food safety perspective, different MRSA strains may have different responses in attachment and biofilm formation *via* food manipulation and/or consumption. This study aimed at evaluating the biofilm forming ability of MRSA strains from food products by an *in vitro* approach. A correlation between biofilm formation, their composition and molecular aspects of MRSA strains has been highlighted.

## Materials and Methods

### Methicillin-Resistant *Staphylococcus aureus* Strains

Forty-nine MRSA strains from foods of animal origin (42 from milk and dairy products and 7 from meat and meat products; Rodríguez-Lázaro et al., 2015, 2017) were used to test their biofilm-forming capacity. The genetic background of all strains has been previously characterized based on MLST, SCC*mec* typing and PFGE typing (Rodríguez-Lázaro et al., 2017). The antimicrobial susceptibility to 20 antimicrobials using a Microscan automated system has been performed following the recommendations of EUCAST guidelines (Rodríguez-Lázaro et al., 2017). Prior to biofilm assays, MRSA strains were transferred from freeze-dried cultures (in 25% glycerol, -80°C) to Baird Parker (BP) agar plates (Biolife Italiana, Milano, Italy), followed by incubation for 24 h at 37°C. Reference strain ATCC 25923 (Stepanović et al., 2000; Bauer et al., 2013) was used as positive control in all assays.

### Biofilm Formation and Quantification

Before use, one single colony of MRSA strains from BP plates into a test tube containing 10 mL of Tryptic Soy Broth (TSB) medium (Oxoid Ltd., Hampshire, England) and incubated for 18 h at 37°C. The inocula of each MRSA strains were adjusted to optical density (OD)_600_ = 1, and confirmed by plate counts of 10-fold dilutions of each MRSA inoculum. These bacterial cultures contained approximately 10^9^ cfu/mL. Experiments in polystyrene microtiter plates were performed as previously described (Oniciuc et al., 2016), using 96-well polystyrene microtiter plates (Nunc^®^ MicroWell^TM^) (Nunc, St. Louis, MO, United States) and using TSB medium enriched with 0.4% (w/v) glucose (TSBG) (Sigma-Aldrich, St. Louis, MO, United States) previously sterilized by filtration on 0.22 μm nitrocellulose filters (Minisart^®^) (Sartorius Stedim Biotech, Germany) and added to the broth media after sterilization. A total volume of 0.2 mL of each broth with a starting inoculum of approximately 10^6^ CFU/mL was added per well, followed by 24 h-incubation at 37°C in static conditions. Crystal violet staining (CV- Merck KGaA, Darmstadt, Germany) was used to quantify the total amount of biofilm biomass attached on the 96-well hydrophobic surfaces (Stepanović et al., 2000). After carefully removal of the broth medium from wells, biofilms were rinsed twice with 0.9% NaCl (Liofilchem, Roseto degli Abruzzi, Italy) to remove weakly adherent cells. Biofilm fixation was achieved using pure methanol (15 min) (Sigma–Aldrich, St. Louis, MO, United States), after discarding the supernatant and allowing the wells to dry at room temperature. Biofilms were stained with 1% CV for 5 min, and then the excess of stain was removed by gently washing with tap water. Absorbance was determined at 570 nm using an ELISA reader (Tecan 900 Pro), after CV bound being released in 0.2 mL of 33% acetic acid (Sigma-Aldrich, St. Louis, MO, United States). *S. aureus* biofilm-forming strain ATCC 25923 (positive control) and broth medium (negative control) were used as controls during all biofilm assays. All experiments were replicated three times on separate days. The cut-off OD (ODc) was defined as three standard deviations above the mean OD of the negative controls. A prolonged incubation time (48 h) was also performed (Peeters et al., 2008) for those MRSA strains exhibiting a biofilm forming capacity higher than OD_570_ 3.

A total of 13 MRSA isolates (those considered as strong biofilm-producers -OD_570_ > 3- in the polystyrene experiments and 4 with a more moderate biofilm formation; i.e., 50, 151-1.1, 153-1.1, 137-2.2, 74.2, 117.2, 117.3, 50-2.1, 74.1, 24.1, 24.2, 46-2.3, and ATCC 25923) were selected to determine their biofilm-forming ability on stainless steel. Biofilm production was measured using the method previously described by Díez-García et al. (2012) with some modifications. Wells of grade 304 stainless steel micro-well plates were filled with 20 μL of the third dilution of this bacterial culture and 180 μL of TSB or TSBG to obtain a concentration of 10^5^ cfu/mL in the well. The negative controls (three in each plate) contained 200 μL of TSB. The plates were incubated aerobically for 24 h at 37°C. The content of the plate was then poured off and the wells washed with 200 μL of distilled water. The bacteria that remained attached were fixed by adding 200 μL of methanol to each well for 15 min. The plates were then emptied, air dried and stained with 200 μL per well of crystal violet for 5 min. Excess stain was rinsed off by placing the micro-well plate under running tap water. The plates were air dried and then the dye bound to the adherent cells was resolubilized with 200 μL of 33% glacial acetic acid per well. The content of the wells was transferred to 100-well polystyrene micro-well plates (Oy Growth Curves Ab Ltd., Helsinki, Finland), and the optical density of each well was measured using a wide band filter (band area 420–580 nm; OD420-580) in a Bioscreen C MBR (Oy Growth Curves Ab Ltd.). The micro-well plates were shaken for 1 min prior to the measurement of turbidity. All experiments were replicated three times on separate days. The cut-off OD (ODc) was defined as three standard deviations above the mean OD of the negative controls. Strains were classified into four categories: not biofilm producers, when OD ≤ ODc, weak biofilm producers, when ODc < OD ≤ (2 × ODc), moderate biofilm producers, when (2 × ODc) < OD ≤ (4 × ODc), or strong biofilm producers, when (4 × ODc) < OD.

### Viable Cell Counts and Dry Weight Determination

#### Viable Cell Counts

After 48 h incubation, non-adherent cells were removed from the 24-well plates and rinsed twice with 0.9% NaCl solution. Then, each biofilm was resuspended in 1 mL of 0.9% saline solution and dislodged by vigorous scraping and vortexing. Viable cell quantification was performed by colony forming units (CFU) counting using BP agar medium. Three replicates of 10-fold dilutions were made and the plates were incubated at 37°C for 24 h, before counting the number of grown colonies using the following equation:

(1) $$CFU/mL=\frac{\sum CFU}{(n_{1}+0.1n_{2})\cdot d}$$

where: ∑ CFU- total number of colonies from plates containing 10–150 colonies;

n_1_- number of plates containing 10–150 colonies each;

n_2_- number of plates from the following dilution (containing no less than 10 colonies);

d- dilution factor corresponding to the first set of plates containing 10–150 colonies.

#### Biofilm Formation

The amount of the biofilm on each well was scraped into 1 mL of 0.9% saline solution. Resuspended biofilm cells were filtered through a preweighted filter (0.45 μm) (Teknokroma, Spain) and air-dried in the oven for 24 h at 105°C. The dry weight (DW) of each biofilm was calculated by the difference between weights. Moreover, the coefficient of variation (e%) was used to estimate the experimental error using the following formula:

(2) $$e\%=\frac{s}{m}\cdot 100$$

where: 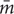
- average of n values;

s- standard deviation of these n values.

### Structural and Matrix Composition Evidenced by Confocal Laser Scanning Microscopy (CLSM)

MRSA cultures were grown at 37°C for 24 h, and twofold dilutions in TSB were made to obtain a concentration of approximately 10^7^ cfu/mL. A volume of 250 μL was added to the wells of Nunc^TM^ MicroWell^TM^ 96-Well Optical-Bottom Plates with Polymer Base (Thermo Fisher Scientific, New Hampshire). After 1 h of adhesion at 37°C, the wells were rinsed with 150 mM of NaCl in order to eliminate any non-adherent bacteria, before being refilled with 250 μL of TSB. After incubation for 24 h at 37°C, the wells were rinsed with 150 mM of NaCl.

Six fluorescent dyes were used. SYTO 9 and propidium iodide (PI) from the LIVE/DEAD^®^ BacLight^TM^ Bacterial Viability Kit, DiIC18(5) oil, 1,1′-dioctadecyl-3,3,3′,3′-tetramethylindodicarbocyanine perchlorate (DiD’oil) and concanavalin A, tetramethylrhodamine conjugate (ConA-TMR) were purchased from Invitrogen (Carlsbad, CA, United States), while fluorescein isothiocyanate isomer I (FITC) and calcofluor white M2R (CFW) were purchased from Sigma (St. Louis, MO, United States).

Five working solutions of stains were prepared in NaCl 150 mM: SYTO 9 (stock 3.34 mM in DMSO) plus PI (stock 20 mM in DMSO) at 1.0 μL/mL each (A), FITC (stock 2 mg in 100 μL absolute ethanol) at 46.6 μg/mL (B), DiD’oil (stock of 25 mg in 2.5 mL of absolute ethanol) at 79.4 μg/mL (C), ConA-TMR (10 mg in 2 mL distilled water with 16.8 mg NaHCO3) at 944.8 μg/mL (D), and CFW at 189 μL/mL (E). To avoid overlapping spectra, a volume of 250 μL of each of the five solutions was added to five different wells (A–E, respectively). The microtiter plate was then incubated in the dark at 37°C. After 25 minwells B, C, D, and E were rinsed with NaCl at 150 mM and refilled with 250 μL of this saline solution.

CLSM image acquisition was performed using a Zeiss LSM 800 Airyscan confocal laser scanning microscope with ZEN 2.3 software (Carl Zeiss, Jena, Germany). Channel mode visualization was done using the 63× (0.8 NA) objective with oil immersion. The microscopic parameters used to study the individual cellular and extracellular components of the biofilms have been previously reported (González-Machado et al., 2018). Three stacks of horizontal plane images (512 × 512 pixels corresponding to 126.8 × 126.8 μm) with a z-step of 0.25 μm, were acquired for each well from three different randomly chosen areas. For image analysis, original Zeiss files (CZI format) were imported into the IMARIS 9.1 software package (Bitplane, Zurich, Switzerland). The individual components of biofilms were represented by fluorescence emitted by SYTO 9 (from cells with intact membranes), PI (from bacteria with damaged membranes), FITC (proteins), DiD’oil (lipids), ConA-TMR (α-polysaccharides), and CFW (β-polysaccharides). Three independent experiments were performed for each MRSA strain.

### Statistical Analysis

Numerical data were compared for statistical significance using analysis of variance techniques (ANOVA) and Duncan’s multiple range test. Significant differences were established at the 5% (*P* < 0.05) level. Data processing was performed using the Statistica^®^ 8.0 software package (StatSoft Ltd., Tulsa, Oklahoma).

## Results

### Biofilm Forming Ability of MRSA Strains: Polystyrene Experiments

**FIGURE 1:**
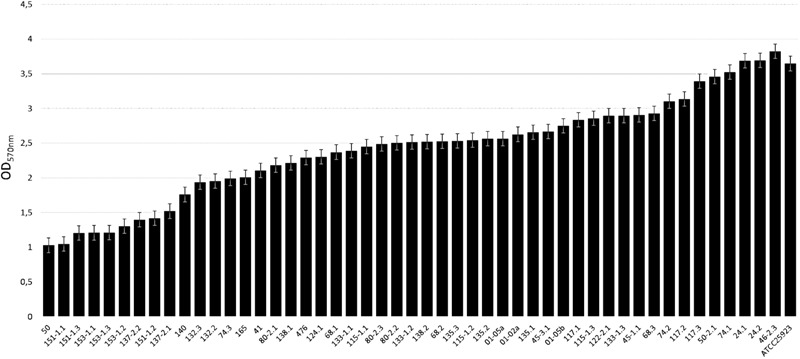
Biofilm forming ability (arranged in increasing order) of MRSA strains isolated from food animal sources using TSBG medium in polystyrene microtiter plates. Bars indicate the average of the OD value ± standard deviation (SD) from three independent experiments.

The MRSA strains were classified in weak (OD_570_ 1.03, SD 0.03), moderate (OD_570_ 1.03–3), and strong (OD_570_ 3.82, SD 0.12) biofilm formers (Figure 1 and Table 1). Forty-one (83.7%) out of the 49 MRSA strains showed moderate biofilm formation, whereas the remaining 8 (16.3%) were strong biofilm producers (Table 1). From the eight strong biofilm producers, 5 were isolated from dairy products (11.90%) and 3 isolated from meat and meat products (42.86%) (Table 2). Overall, a higher biofilm biomass was observed for those MRSA strains harboring SCC*mec* type IV (Table 1), being in accordance with previous studies that proved that such strains harboring SCC*mec* have greater probability to produce higher biofilm biomasses in comparison with those carrying SCC*mec* types I-III (Vanhommerig et al., 2014).

**Table 1:** Biofilm formation pattern in static conditions (24 h) after preenrichment in TSBG by the MRSA strains.

Isolate	Sample type	Country of origin	SCC*mec*	ST	Biofilm formation	
					Polystyrene	Stainless steel
50	Meat	Egypt	IV	22	Moderate	Moderate
151–1.1	Cheese	Bolivia	IV	1649	Moderate	Moderate
151–1.2		Bolivia	IV	1649	Moderate	n.t.
151–1.3		Bolivia	IV	1649	Moderate	n.t.
153–1.1^∗^	Cheese	Bolivia	IV	8	Moderate	Strong
153–1.2^∗^		Bolivia	IV	8	Moderate	n.t
153–1.3^∗^		Bolivia	IV	8	Moderate	n.t
137–2.1	Fresh meat	Republic of Serbia	V	398	Moderate	n.t.
137–2.2					Moderate	Moderate
140	Brined cheese	Turkey	V	5	Moderate	n.t.
132.2	Cheese	Columbia	IV	5	Moderate	n.t.
132.3					Moderate	n.t.
165	Cheese	Turkey	IV	22	Moderate	n.t.
41	Cheese	Egypt	V	5	Moderate	n.t.
80–2.1	Cheese	Nicaragua	IV	5	Moderate	n.t.
80–2.2					Moderate	n.t.
80–2.3					Moderate	n.t.
138.1	Cheese	Nicaragua	IV	5	Moderate	n.t.
138.2					Moderate	n.t.
476	Cheese in spicy sauce	Egypt	V	1	Moderate	n.t.
124.1	Cheese	Nicaragua	IV	5	Moderate	n.t.
68.1	Cheese	Nicaragua	V	72	Moderate	n.t.
68.2					Moderate	n.t.
68.3					Moderate	n.t.
133–1.1	Cheese	Nicaragua	IV	5	Moderate	n.t.
133–1.2					Moderate	n.t.
133–1.3					Moderate	n.t.
115–1.1	Cheese	Bolivia	IV	1649	Moderate	n.t.
115–1.2					Moderate	n.t.
115–1.3					Moderate	n.t.
01–05a	Maturated sheep cheese	Romania	IV	1	Moderate	n.t.
01–05b					Moderate	n.t.
01–02a	Soft goat cheese	Romania	IV	1	Moderate	n.t.
135.1	Cheese	Peru	IV	1649	Moderate	n.t.
135.2					Moderate	n.t.
135.3					Moderate	n.t.
45–3.1	Fresh beef meat	Egypt	V	5	Moderate	n.t.
117.1^∗^	Cheese	Ecuador	IV	8	Moderate	n.t.
117.2^∗^					Strong	Strong
117.3^∗^					Strong	Strong
122–2.1^∗^	Cheese	Peru	IV	8	Moderate	n.t.
45–1.1	Cheese	Egypt	V	97	Moderate	n.t.
50–2.1	Dried meat	China	IV	7	Strong	Moderate
74.1	Cheese	Bolivia	IV	1649	Strong	Strong
74.2					Strong	Strong
74.3					Moderate	n.t.
24.1	Sheep meat	Nigeria	V	8	Strong	Strong
24.2	Sheep meat	Nigeria	V	8	Strong	Moderate
46-2.3	Cheese	Republic of Honduras	IV	7	Strong	Moderate

*^∗^Presence of pvl gene. n.t., non-tested strains.*

**Table 2:** Biofilm formation by MRSA strains in 24 h on hydrophobic 96-well microtiter plates at 37°C, static conditions.

Source	Biofilm producer		Moderate biofilm producer		Strong biofilm producer	
	n	%	n	%	n	%
Milk and dairy products	42	85.7	37	75.5	5	10.2
Meat and meat products	7	14.3	4	8.17	3	6.13

**FIGURE 2:**
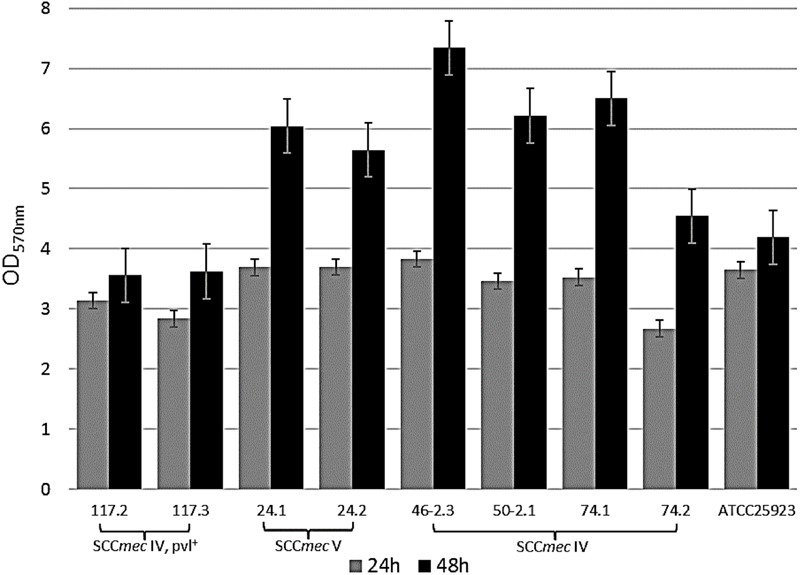
Quantification of 24 and 48 biofilm biomasses using TSBG. Bars indicate the average of the OD value ± standard deviation (SD) from three independent experiments. Negative samples have been extracted from the shown values.

**FIGURE 3:**
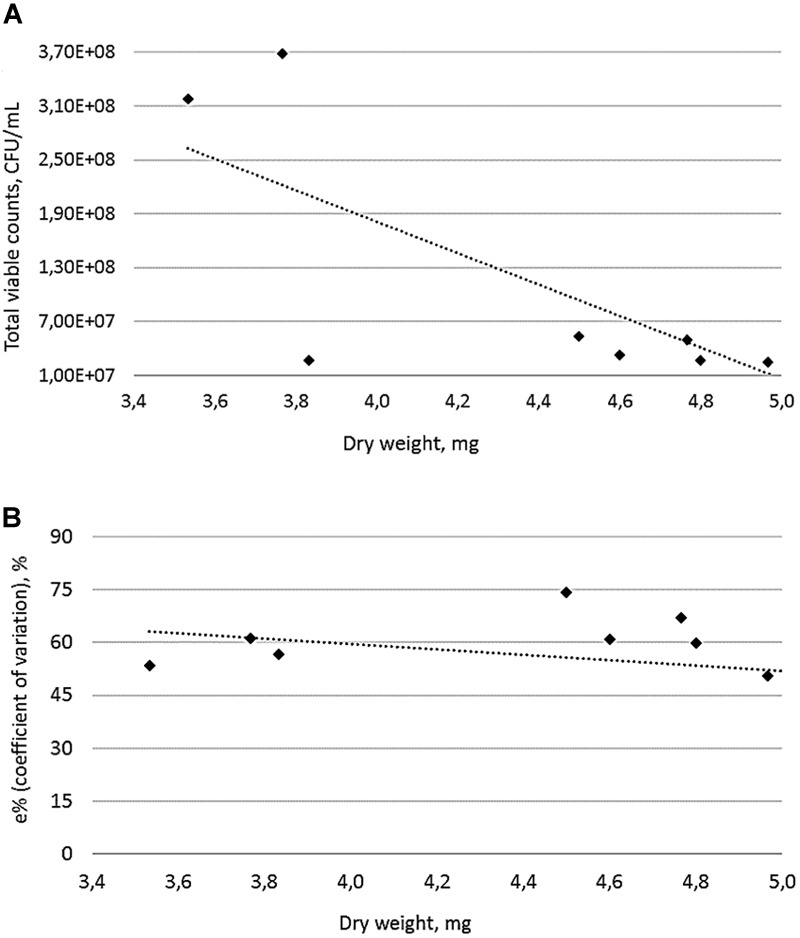
Linear representation (dotted line) between biomass and viable cell quantification of 48 h MRSA biofilm formers (plotted dots). Values indicate the average ± standard deviation from three independent experiments **(A)** and Coefficient of variation as a function of biofilm biomass in MRSA strains **(B)**. In both figures, linear regression lines are shown as a dotted lines.

A prolonged incubation period (48 h) was applied for those eight MRSA strains with an OD_570_ > 3, resulting in an increased biofilm biomass accumulation during the time course (Figure 2). Based on these findings, we further characterized MRSA strains after 48 h of incubation. Similar to the OD measurements, significant differences in viable cell quantification and dry weight calculations of MRSA strains were observed for the 48 h biofilms. The number of viable cells within a MRSA-producing biofilm decreases as its biomass increases (Figure 3A), while the coefficient of variation calculated for the 48 h biofilms biomasses shows a low error (less than 15%) between the different plotted biomasses (Figure 3B). This may be explained by the fact that the metabolic activity, can differ as bacterial cells from biofilms are competing for nutrients available in a limited space. The remaining viable cells may show different metabolic states depending on the total biofilm biomass accumulated during a 48 h period. Although *Staphylococcus* spp. are known to produce strong biofilms (Bridier et al., 2010), this was not the case for the biofilm mode of growth of the MRSA strains 117.2 and 117.3, in which cell viability was higher, explained by the lack of cells adhesion to the polystyrene surfaces.

### Biofilm Forming Ability of MRSA Strains: Stainless Steel Experiments

**FIGURE 4:**
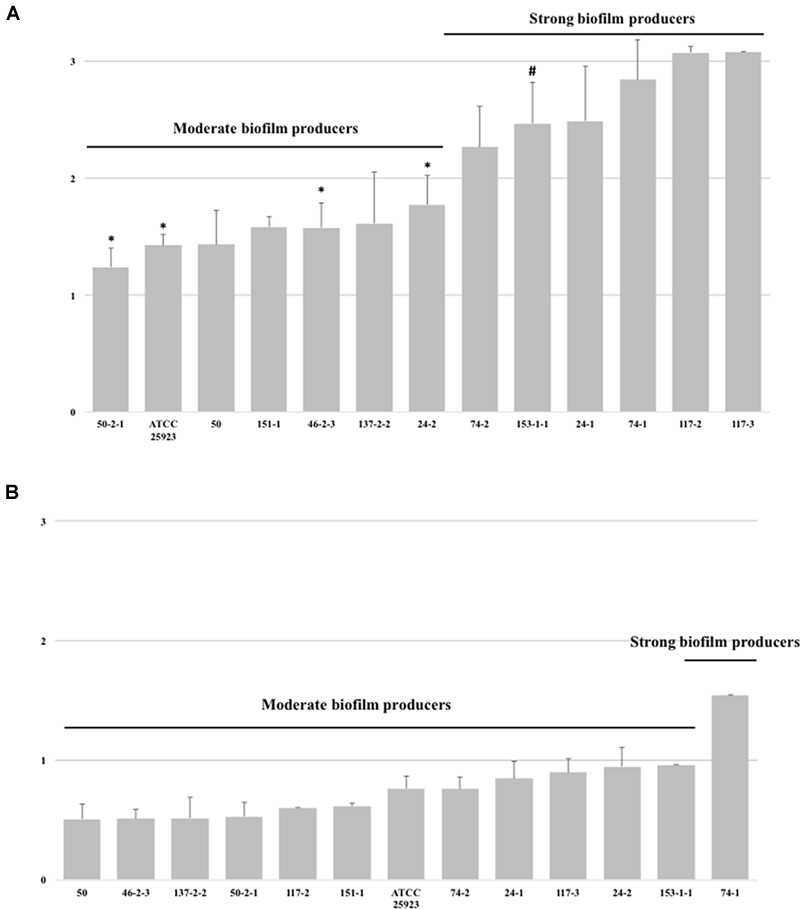
Biofilm forming ability (arranged in increasing order) of MRSA strains isolated from food animal sources in stainless steel micro-well plates using TSB broth supplemented **(A)** or not **(B)** with glucose. Bars indicate the average of the OD value ± standard deviation (SD) from three independent experiments. ^∗^Indicates strains that were strong biofilm producers in polystyrene microtiters plates, but moderate biofilm producers in stainless steel. ^#^Indicates strains that were moderate biofilm producers in polystyrene microtiters plates, but strong biofilm producers in stainless steel.

The capacity for producing biofilm of the MRSA strains in stainless steel was tested using the MRSA strains with a previous evidence of strong production of biofilm in polystyrene microtiter plates and in a subset of MRSA strains with a moderate capacity for producing biofilms (Figures 4A,B). Interestingly, biofilm formation was significantly different if the MRSA strains were pre-enriched in TSB supplemented or not with glucose. MRSA strains pre-enriched in TSBG were clustered in two categories; moderate and strong biofilm producers with an average OD_570_ of 1.52 ± 0.26 and 2.69 ± 0.42, respectively (Figure 4A). However, lower OD_570_ readings were observed when MRSA strains were pre-enriched in TSB (not supplemented with glucose), and were clustered in three categories; weak, moderate and strong biofilm producers with an average OD_570_ of 0.55 ± 0.10, 0.86 ± 0.13, and 1.54 ± 0.00, respectively (Figure 4B). Likewise, in all the MRSA strains tested in stainless steel regardless the addition or not the glucose during the pre-enrichment, the OD values were significantly lower than those observed in biofilm in polystyrene microtiter plates. In addition, the capacity of biofilm production correlated pretty well between the experiments using polystyrene microtiter plates and stainless steel micro-well plates (using TSBG as pre-enrichment broth); only 1 (MRSA strain 153.1-2) out of 4 of the MRSA strains observed as moderate biofilm producers in polystyrene microtiter plates, showed a stronger capacity of biofilm production in stainless steel, while 3 (MRSA strains 50.2-1, 24.2, and 46.2-3) of 8 the MRSA strains observed as strong biofilm producers in polystyrene microtiter plates were classified as moderate biofilm producer. Overall, as observed in polystyrene microtiter plates a higher biofilm production was found for those MRSA strains harboring SCC*mec* type IV.

### Biofilm Determination by Confocal Laser Scanning Microscopy (CLSM)

**FIGURE 5:**
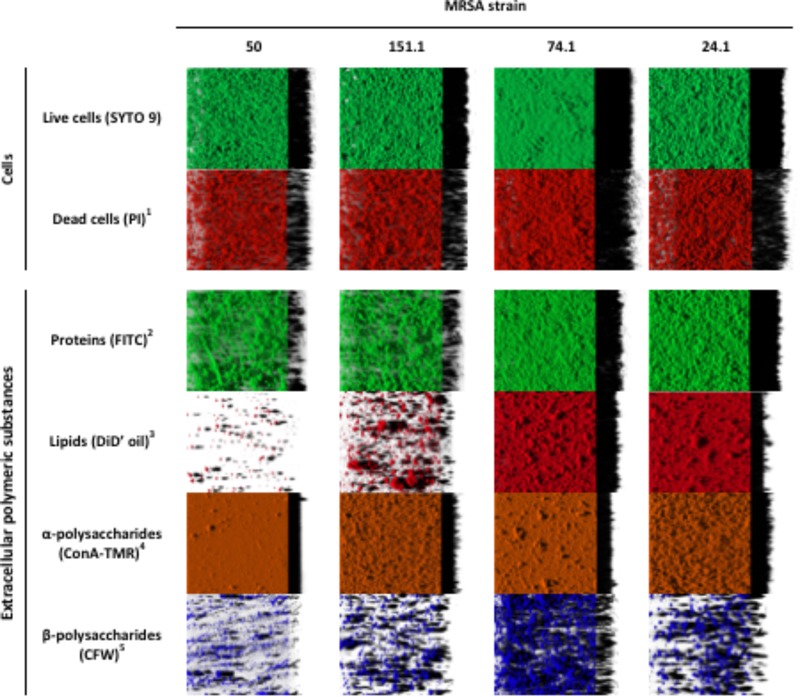
Three-dimensional projections of individual components in biofilms formed by four methicillin-resistant *Staphylococcus aureus* (MRSA) strains on polystyrene after 24 h of incubation. The images were obtained from confocal stack images by the IMARIS 9.1 software, virtual projections being included on the right. ^1^propidium iodide; ^2^fluorescein isothiocyanate isomer I; ^3^DiIC18(5) oil, 1,1′-dioctadecyl-3,3,3′,3′-tetramethylindodicarbocyanine perchlorate; ^4^concanavalin A, tetramethylrhodamine conjugate; ^5^calcofluor white M2R.

The structural parameters of individual cellular and extracellular components of biofilms formed by four MRSA strains were evaluated by CLSM observations and digital image analysis. Table 3 shows biovolume values of each of the biofilm components after 24 h of incubation at 37°C. Representative three-dimensional renderings of individual components for the strains under study are presented in Figure 5.

**Table 3:** Biovolume (mean ± SD) of individual components in biofilms formed by four methicillin-resistant *Staphylococcus aureus* (MRSA) strains on polystyrene after 24 h of incubation.

		MRSA strain			
		50	151.1	74.1	24.1
Cells			
	Live cells (SYTO 9)	93,443.9 ± 2,061.3^a^_e_	125,409.0 ± 2,943.1^b^_d_	296,871.0 ± 4,846.3^d^_f_	258,637.8 ± 22,514.8^c^_e_
	Dead cells (PI)^1^	34,575.1 ± 2,035.9^b^_d_	29,031.8 ± 771,6^a^_b_	85,657.8 ± 2,409.5^d^_b_	46,172.5 ± 3,508.6^c^_b_
Extracellular polymeric substances			
	Proteins (FITC)^2^	29,349.8 ± 1,839.6^a^_c_	28,756.0 ± 3,772.6^a^_b_	118,424.5 ± 2,069.1^b^_d_	235,171.0 ± 6,391.1^c^_e_
	Lipids (DiD’ oil)^3^	1,545.9 ± 55.0^a^_a_	20,077.0 ± 824.7^b^_a_	123,598.7 ± 2,519.1^d^_e_	92,528.3 ± 5,710.5^c^_c_
	α-polysaccharides (ConA-TMR)^4^	103,921.3 ± 5,826.0^b^_f_	118,132.7 ± 3,093.4^c^_c_	89,345.4 ± 2,200.4^a^_c_	145,085.6 ± 4,283.1^d^_d_
	β-polysaccharides (CFW)^5^	6,221.6 ± 328.8^a^_b_	18,446.1 ± 663.6^b^_a_	58,251.8 ± 1,251.7^d^_a_	24,814.5 ± 4,035.4^c^_a_

*Data (n = 9) in the same row with no superscript letters in common are significantly different (P < 0.05). Data in the same column with no subscripts letters in common are significantly different (P < 0.05). ^*1*^propidium iodide; ^*2*^fluorescein isothiocyanate isomer I; ^*3*^DiIC18(5) oil, 1,1′-dioctadecyl-3,3,3′,3′-tetramethylindodicarbocyanine perchlorate; ^*4*^concanavalin A, tetramethylrhodamine conjugate; ^*5*^alcofluor white M2R.*

**FIGURE 6:**
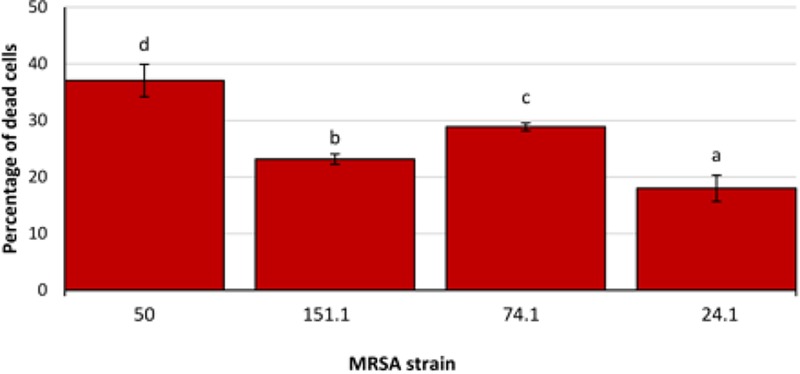
Percentage of dead cells relative to total cells in biofilms formed by four methicillin-resistant *Staphylococcus aureus* (MRSA) strains on polystyrene after 24 h of incubation. Bars (mean ± SD; *n* = 9) with no letters in common are significantly different (*P <* 0.05).

Significant differences were observed between strains, with a lower (*P* < 0.05) biovolume for most individual components in MRSA 50 and MRSA 151.1 biofilms as compared with biofilms formed by MRSA 74.1 and MRSA 24.1. Biovolume of life (SYTO 9 stained) cells in the observation field (16,078.2 μm^2^) ranged from 93,443.9 ± 2,061.3 μm^3^ (strain 50) to 296,871.0 ± 4,846.3 μm^3^ (strain 74.1). Staining the biofilm with propidium iodide (PI) showed the presence of red aggregates of materials probably formed by a mixture of dead or damaged cells and extracellular DNA (eDNA). The percentage of dead cells (PI-stained) relative to total cells ranged from 18.00 ± 2.31% for strain 24.1–37.05 ± 2.86% for strain 50 (Figure 6).

Proteins and α-polysaccharides (FITC- and ConA-TMR-stained compounds, respectively) showed the greatest biovolume among the extracellular components within the biofilms, with values ranging from 28,756.0 ± 3,772.6 μm^3^ (strain 151-1) to 235,171.0 ± 6,391.1 μm^3^ (strain 24.1) for proteins, and from 89,345.4 ± 2,200.4 μm^3^ (strain 74.1) to 145,085.6 ± 4,283.1 μm^3^ (strain 24.1) for α-polysaccharides. On the other hand, analysis of CLSM images revealed low biovolumes for fluorescent labeled lipids (stained with DiD’oil, these lying between 1,545.9 ± 55.0 μm^3^ for strain 50 and 123,598.7 ± 2,519.1 μm^3^ for strain 74.1) and β-polysaccharides (stained with CFW; ranging between 6,221.6 ± 328.8 μm^3^ for strain 50 and 58,251.8 ± 1,251.7 μm^3^ for strain 74.1). The greater the biovolume of biofilms, the more abundant were components stained with FITC, the dye for proteins, and with DiD’ oil, that for lipids. On the other hand, biovolumes of α-polysaccharides and β-polysaccharides were scarcely influenced by biomass of biofilm.

## Discussion

Bacterial ability to form biofilms is of great importance and represents a big challenge for the food industry, as some strains in their sessile state may tolerate antimicrobial agents, making the bacterium extremely difficult to eradicate (Basanisi et al., 2017). Together with *S. epidermidis*, MRSA has been extensively studied in clinical settings due to its capacity to adhere to catheters, indwelling medical devices or to colonize different surfaces (Ferreira et al., 2012; Prakash et al., 2016). The emergence of *S. aureus* resistant to antimicrobial agents has provoked considerable concern due to its presence in associated foodstuffs (Rodríguez-Lázaro et al., 2015). MRSA could adhere and form biofilms on different surfaces used widely in the food sector such as polystyrene, stainless steel, glass, ceramic and other (Oulahal et al., 2008; Mirani et al., 2013; Di Ciccio et al., 2015; Vergara et al., 2017; Rodríguez-Melcón et al., 2018). There is also a risk that MRSA could produce biofilms by contamination from human handlers rather than from food itself (Doulgeraki et al., 2017).

In the present study, forty-nine MRSA from food sources were tested for their biofilm formation ability using TSBG media at 37°C. TSB supplemented with glucose or NaCl has been shown to improve the biofilm formation on microtiter plates as suggested by some researchers in their attempt to find the best culture media in which *S. aureus* may be able to form reproducible and robust biofilms (Luong et al., 2009; Merino et al., 2009; Chen et al., 2012; Oniciuc et al., 2016). Our results on stainless steel are in agreement with those previous publications as we observed a significant reduction on the OD values in all the strain tested in stainless steel when pre-enriched with TSB not supplemented with glucose. This can be relevant from a food safety perspective, as if the cleaning procedures are not implemented correctly, glucose residues can remain on the surfaces from the environment of food facilities, and become a triggering factor for MRSA biofilm production.

Our results show a variation in the ability to form biofilms based on OD measurements: most of the analyzed MRSA strains had the ability to form moderate (83.7%) biofilms on the surface of polystyrene microtiters plates, but there are also strains capable of forming strong biofilms (16.3%). Interestingly, the capacity of biofilm formation was similar on stainless steel; although lower OD values were observed, most of the MRSA strains retained the strong biofilm capacity on this type of material, which is widely used in the food industry. Hydrophobicity seems to be an important factor contributing to the biofilm formation capacity of MRSA strains, in accordance with previous studies (Pagedar et al., 2010). In this study, the most common SCC*mec* type was IV/ST5, followed by the SCC*mec* type IV/ST8 harboring the *pvl* gene. The latter suggest human handling as being the prime source of food contamination (Ogata et al., 2012; Rodríguez-Lázaro et al., 2015).

Evaluation of 48 h MRSA-biofilms has been achieved by correlating the number of viable cells within the total amount of biofilm biomass. Based on DW measurements, the strain MRSA 74.1 had a significantly higher biomass than the biofilm-producing model ATCC 25923, but those differences were not correlated with the CFU counting after 48 h of growth. This may be explained by the fact that MRSA strain 74.1 accumulated a denser biofilm matrix, with the remaining cells competing for survival. However, older biofilms may have stable cell clusters which can interfere with the quantification of sessile bacteria by plate-counting (Freitas et al., 2014). Different distribution in cell densities well as the self-produced extracellular polymeric matrix were observed. In addition, CLSM allowed a visual analysis of the concurrent distribution of polysaccharides, nucleic acids, and proteins components within the biofilms. Our results revealed that that MRSA strains isolated from foods have significant capacity for forming biofilms with a higher protein content confirming that protein-based matrices are of prime importance for the structure of biofilms formed by MRSA. This finding could be very useful for defining more rationale hygienic protocols in the food industry as well as to define more fit-for-purpose anti-biofilm compounds.

In this study, different biofilm patterns related to MRSA clonal lineages were observed, especially for those harboring SCC*mec* type IV and V, in accordance with observations made by other studies. For example, Mirani et al. (2013) found that 98.3% of MRSA strains harbored SCC*mec* type IV, which was related to their biofilm ability. Moreover, different biofilm patterns related to MRSA clonal lineages has been observed: MRSA strains harboring SCC*mec* type IV produce significantly more biomass under static conditions than SCC*mec* type I–III but better biofilm formers are associated to SCC*mec* type I–III when dynamic conditions are used (Vanhommerig et al., 2014). However, Parisi et al. (2016) observed an association between SCC*mec* type IV or V and biofilm formation, whereas the high prevalence of such staphylococcal cassettes promotes *S. aureus* biofilm producing ability, thus allowing the bacterium to persist in the environment.

CLSM together with quantitative image analysis allows for the determination of structural parameters, permitting quantitative comparison of biofilms from different MRSA strains. In agreement with previous research (Buzón-Durán et al., 2017; Rodríguez-Melcón et al., 2018), MRSA strains were able to form compact biofilms on polystyrene after 24 h of incubation. This finding is a matter of concern because plastic materials are frequently used in the installations and equipment of food-processing facilities and the medical system. Because bacteria within a biofilm are typically more resistant to antimicrobials than planktonic (free-living) microbes, the results in this study underline how crucial it is to sanitize food equipment and medical devices immediately after use to prevent biofilm formation.

Percentages of PI-stained (red) cells detected in this research were higher than those previously observed for biofilms grown on polystirene after 24 h of incubation, where values of 1.46 ± 1.15% (Rodríguez-Melcón et al., 2018) or 10.16 ± 4.04% (Buzón-Durán et al., 2017) have been reported. Because the ability to produce biofilms can vary among isolates within a species, it is likely that different results can be obtained using different bacterial strains (Díez-García et al., 2012). The origin of the eDNA composing the biofilms remains unclear but might involve cellular lysis as a consequence of programmed cell death associated with the life-cycle of bacteria or the release of small vesicles. It has been reported that eDNA has a key role in intercellular adhesion and biofilm stability, promoting biofilm formation (Rodríguez-Melcón et al., 2018).

The control of biofilms does not necessarily require the direct killing of the bacteria in the biofilm but might be directed toward the dispersal of degradation of the extracellular polymeric matrix. In such a scenario, a better understanding of the organization and development of the matrix within biofilms is essential for devising effective strategies for the control and eradication of these structures. In the present study, proteins and α-polysaccharides were the predominant components inside the extracellular polymeric matrix of MRSA strains, whereas lipids were important components only for strong biofilm producer strains, and a low biovolume of β-polysaccharides was observed for all strains. Other authors have also shown proteinaceous components to be the key components of the MRSA biofilm matrix (Dakheel et al., 2016).

## Conclusion

In conclusion, the capacity to form biofilms can be a key aspect for the potential role of food in the successful dissemination of MRSA lineages, as it could act as a survival strategy against harsh environmental conditions. Our results confirm that MRSA strains isolated from foods of animal origin have a significant capacity to form biofilms with a significant protein content confirming that protein-based matrices are of prime importance for the structure of biofilms formed by MRSA, and may be used to identify MRSA biofilm formation. The knowledge of the capacity of MRSA strains to produce biofilms, as well as the characterization of the main MRSA biofilms matrix components can help to counteract the mechanisms involved in biofilm resistance, and define more accurate control strategies, such as the use of biofilm-degrading enzymes, quorum sensing inhibitors or bacteriophages.

## Author Contributions

DR-L designed, supervised the experiments, analyzed the results, revised the first draft, and prepared the last draft of the manuscript. CA-C performed part of the experiments, supervised the experiments, analyzed the results, and revised the last draft of the manuscript. EO performed part of the experiments, analyzed the data and prepared the first draft of the manuscript. RC, VB, DG, and CG-M performed part of the experiments. MH, MW, and AN collaborated in the design of the experiments and revised the different versions of the manuscript. JE-B collaborated in the design of the experiments, in the supervision of the experiments, and in the analysis of the results and revised the different versions of the manuscript.

## Conflict of Interest Statement

The authors declare that the research was conducted in the absence of any commercial or financial relationships that could be construed as a potential conflict of interest.
